# DNA Binding Characteristics and Protective Effects of Yellow Pigment from Freshly Cut Yam (*Dioscorea opposita*)

**DOI:** 10.3390/molecules25010175

**Published:** 2020-01-01

**Authors:** Lei Zhao, Xiaoyan Zhao, Yue Ma, Yan Zhang, Dan Wang

**Affiliations:** 1 Beijing Vegetable Research Center, Beijing Academy of Agriculture and Forestry Science, Beijing Key Laboratory of Agricultural Products of Fruits and Vegetables Preservation and Processing, Key Laboratory of Vegetable Postharvest Processing, Ministry of Agriculture and rural affairs, Beijing 100097, China; zhaoleibvrc@163.com (L.Z.); mayue@nercv.org (Y.M.); 2 College of Food Science, Shenyang Agricultural University, Shenyang 110866, China; 3 Longda Food Group Company Limited, Shandong, Jinan 265231, China

**Keywords:** freshly cut yam, yellow pigment, calf thymus DNA, protection, DNA damage

## Abstract

Yam yellow pigments (YP) are natural pigments formed during the storage of freshly cut yam (*Dioscorea opposita*) under certain conditions. The interaction of YP with calf thymus DNA (ctDNA) and its protective effect against DNA oxidative damage were investigated using multiple spectroscopic techniques, competitive binding experiments, viscosity measurements, and gel electrophoresis. Results showed that YP participated in intercalative binding with ctDNA. YP exhibited a protective effect against hydroxyl-induced DNA damage, which was attributed to the high hydroxyl radical scavenging activity of YP. Our findings improve our understanding of the mechanism of interaction between YP and ctDNA, and provide a theoretical basis for the application of YP in the food and drug industry.

## 1. Introduction

DNA is a remarkable genetic material found in living organisms [1]. The bases and sugar moieties in DNA are easily attacked by excessive reactive oxygen species [2], and enzymatic repair may not always be sufficient to protect against permanent DNA damage [3]. Scientists in the fields of chemistry, life science, medicine, and structural biology have all studied how to best protect DNA from damage [4]. Many clinically used drugs realize their therapeutic effects by interfering with the processes of DNA copying and transcription. Various flavonoids and pigment-containing extracts have been shown to protect DNA from damage. For example, apigenin protects plants against damage induced by ultraviolet (UV)-B radiation [5]; Chinese bayberry extract protects against oxidative DNA damage [6]; grape-seed extract prevents oxidation-induced DNA damage [7]; and cyanidin-3-glucoside, along with its aglycon, protect against calf thymus DNA (ctDNA) damage [8].

Fresh-cut yam (*Dioscorea opposita*) slices are easy to brown, but they became yellow under certain storage conditions in our previous experiments. Yellow pigment (YP) is composed primarily of bisdemethoxycurcumin (73.7%)—a kind of naturally formed pigment similar to anthocyanin and carotene—and two other unknown compounds with formulae C_19_H_16_O_3_ and C_19_H_18_O_3_ [9]. The ability of YP to combine with ctDNA, the binding mode to ctDNA, and the protective effect on ctDNA have never been reported. Therefore, the DNA-binding characteristics and in vitro protective ability of YP against ctDNA damage were investigated using a series of spectroscopic methods, along with measurements of DNA viscosity and hydroxyl radical (∙OH) scavenging activity. The findings deepen our understanding of the binding mechanism of YP with ctDNA, and provide a basis for applying YP in the food industry and in the development of biosimilars.

## 2. Results

### 2.1. YP Binding to DNA

**Figure 1:**
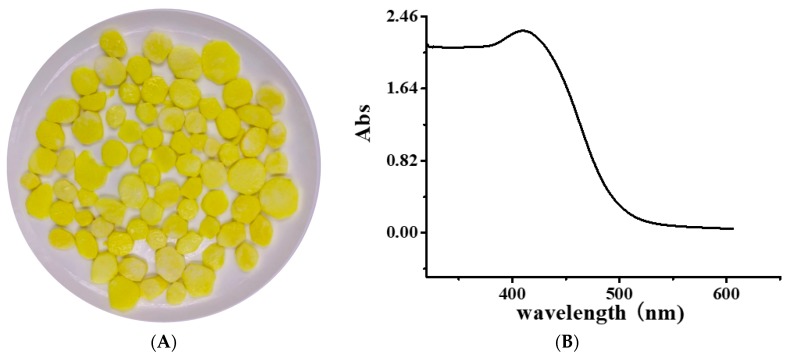
(**A**) Image of yellow freshly cut yam and (**B**) Ultraviolet-Visible (UV–Vis) spectrum of yellow pigment (YP).

**Figure 2:**
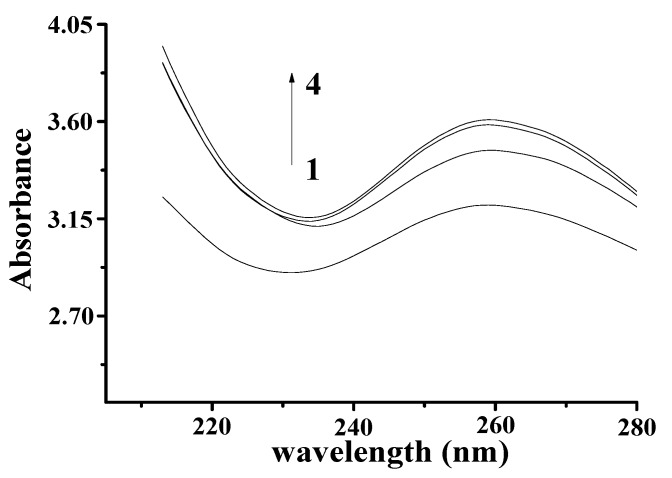
Absorption spectra of YP–DNA (C_YP_ = 0.9 mg/mL, C_DNA_ = 4 mg/mL) upon addition of increasing volume: (1) 0 μL, (2) 50 μL, (3) 60 μL, (4) 70 μL.

The Ultraviolet-Visible (UV–Vis) spectrum of YP is shown in Figure 1B. The obvious absorption peak at 410 nm corresponds to the typical yellow absorption band. The UV–Vis spectra of DNA and the DNA–YP mixtures are compared in Figure 2. Absorption intensity increased upon the addition of YP, and it increased gradually with increasing YP content. This indicates that the conformation of DNA was affected by electrostatic interactions between the basic groups of DNA and the YP [10,11,12]. Furthermore, a bathochromic shift was observed in the maximum absorption of the DNA–YP mixtures. Bathochromic effects were also observed in the visible absorption (Figure 2), providing clear evidence for the binding between YP and ctDNA. Similarly, the binding of the novel 9-O-N-aryl/aryl-alkyl amino carbonyl methyl to ctDNA [13] and the formation of complexes between DNA and fluorenylidene double-bridged cyclotriphosphazene derivatives [14] were found to contribute to the bathochromic shift of visible absorption, which thus supported the formation of DNA–YP complexes.

### 2.2. Binding Mode of DNA–YP

**Figure 3:**
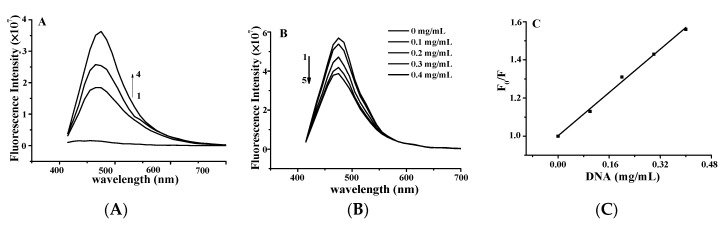
(**A**) Fluorescence quenching spectra of DNA–YP complexes (*C*_DNA_ = 0.9 mg/mL) upon addition of different YP concentrations: (1) 0 mg/mL, (2) 0.9 mg/mL, (3) 1.1 mg/mL, and (4) 1.3 mg/mL. (**B**) Effect of DNA on YP fluorescence spectrum. (**C**) Stern–Volmer plot for binding of YP by DNA at room temperature.

The effect of YP on the fluorescence spectrum of ctDNA is shown in Figure 3A. The fluorescence intensity of DNA increases when small molecules are intercalated into the nitrogenous bases of DNA [15,16]. The fluorescence intensity of DNA increased with increasing YP concentration, further confirming the binding between YP and DNA. Figure 3B shows the effect of DNA on the fluorescence spectrum of YP. YP exhibited a strong fluorescence emission peak at 475 nm under excitation at 380 nm, and intensity decreased gradually with increasing DNA concentration.

The Stern–Volmer equation was applied to evaluate the binding constant of the biomolecular quenching process (*K*_q_) as follows: *F*_0_/*F* = 1 + *K*_q_*τ*_0_[DNA] = 1 + *K*_sv_[DNA] [17], where *F*_0_ and *F* are the fluorescence intensities in the absence and presence of ctDNA in free YP, [DNA] is the concentration of ctDNA, and *τ*_0_ is the average fluorescence lifetime of YP (10^−8^ s). *K*_q_ was calculated to be 1.426 ± 0.12 × 10^8^ L·mol^−1^·s^−1^ on the basis of the intercept-to-slope ratio of the plot of *F*_0_/*F* vs. [DNA] (Figure 3C; R^2^ = 0.9993). *K_s_*_v_ was evaluated by the linear fitting of data in terms of the above equation.

In general, *K*_q_ values for quenching agents of biomolecules are no more than 2 × 10^10^ L·mol^−1^·s^−1^ [18]. In this study, the calculated *K*_q_ of YP was lower than that of the diffusion control constant (2 × 10^10^ L·mol^−1^·s^−1^), confirming that the quenching was caused by dynamic collision rather than groove binding [19]. This further suggests that the binding between YP and ctDNA was intercalative in nature.

As a planar phenazine dye, neutral red (NR) has a similar structure to other planar dyes (e.g., acridine, thiazine, xanthene). The interaction of fluorescent NR with DNA has been confirmed by spectrophotometric [20] and electrochemical [21] techniques. NR intercalates into the base pairs of double-helix DNA [22] and exhibits low toxicity and good stability (up to two years). Thus, NR was applied as a molecular probe to evaluate the intercalation of YP.

**Figure 4:**
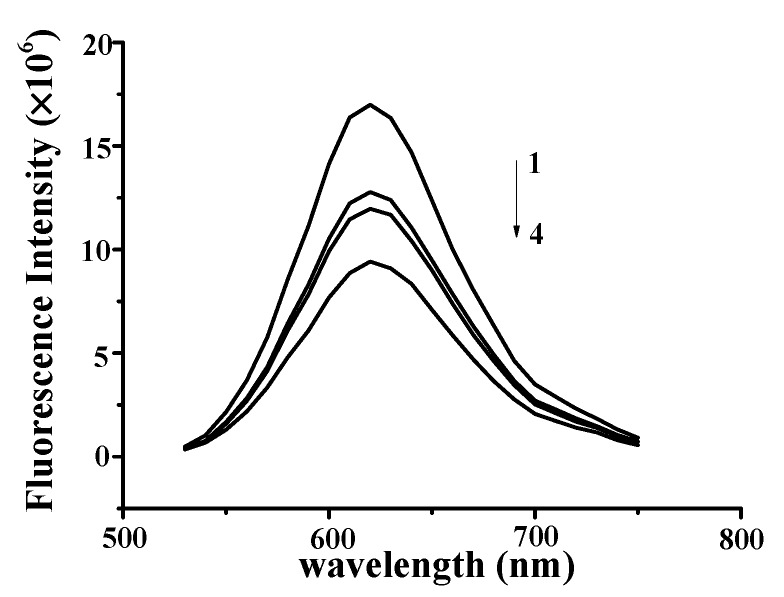
Fluorescence quenching spectra of DNA–YP–NR complexes (*C*_DNA_ = 0.9 mg/mL, *C*_NR_ = 5 × 10^−3^ mg/mL) upon the addition of different YP concentrations: (1) 0 mg/mL, (2) 0.55 mg/mL, (3) 0.65 mg/mL, and (4) 0.75 mg/mL.

The observed fluorescence intensity of the NR–DNA complexes gradually decreased with increasing YP concentration from 0 to 0.75 mg/mL (Figure 4), indicating that YP intercalated into the DNA by substituting for NR in the NR binding sites. These findings confirmed that YP was bound to DNA through intercalation [23,24]. Similar results were reported for butylated hydroxyanisole and ctDNA [25]. Results verified that strong intercalative binding occurred between YP and DNA to form DNA–YP complexes.

### 2.3. Binding of YP to DNA in the Presence of Competing Ions

Since DNA is an anionic polyelectrolyte with phosphate groups, it prefers to bind cations through electrostatic interactions [26]. Binding modes between chemical compounds and DNA can be distinguished by spectroscopic changes upon variation in ionic strength. Sodium ions (Na^+^) were shown to weaken the electrostatic interactions between chemical compounds and DNA by competing with the phosphate groups of DNA due to the anionic phosphoric acid groups already present on the DNA backbone [27,28]. Thus, sodium chloride (NaCl) was chosen as a competitive ionic reagent in this study.

**Figure 5:**
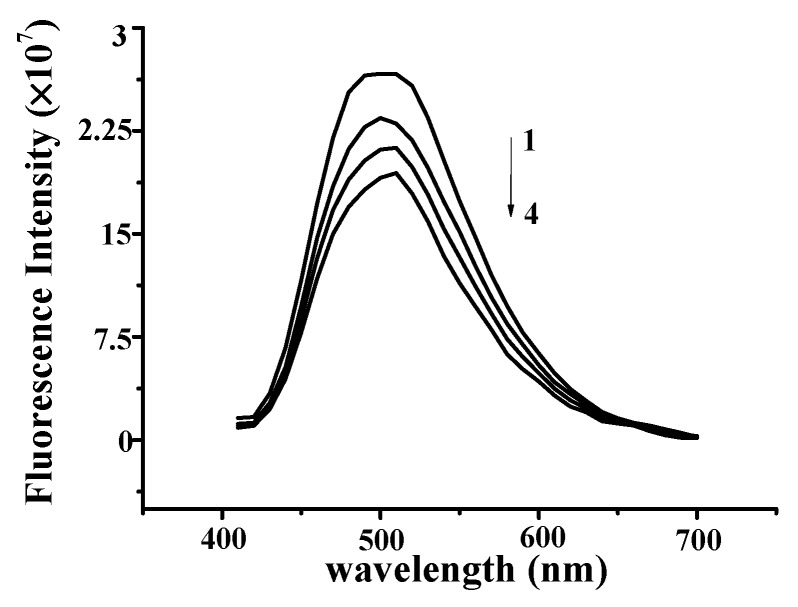
Cationic fluorescence quenching spectra of DNA–YP (*C*_DNA_ = 0.9 mg/mL, *C*_YP_ = 1.5 mg/mL) upon the addition of different concentrations of NaCl: (1) 0 mg/mL, (2) 0.2 mg/mL, (3) 0.4 mg/mL, and (4) 0.6 mg/mL.

As shown in Figure 5, the fluorescence intensity of DNA–YP decreased as NaCl concentration increased, indicating that the addition of NaCl weakened the electrostatic binding of YP and DNA. This further demonstrates that YP interacted electrostatically with DNA.

### 2.4. DNA Viscosity

Viscosity measurements can be used to evaluate DNA length changes and provide information about binding modes between small molecules and DNA [29]. When small molecules intercalate into the DNA helix, the DNA must lengthen to allow the binding of ligands, resulting in a significant increase in DNA viscosity [30]. In the case of the partial and/or nonclassical intercalation of small-molecule ligands in DNA, no effect on DNA viscosity is observed [31,32].

**Figure 6:**
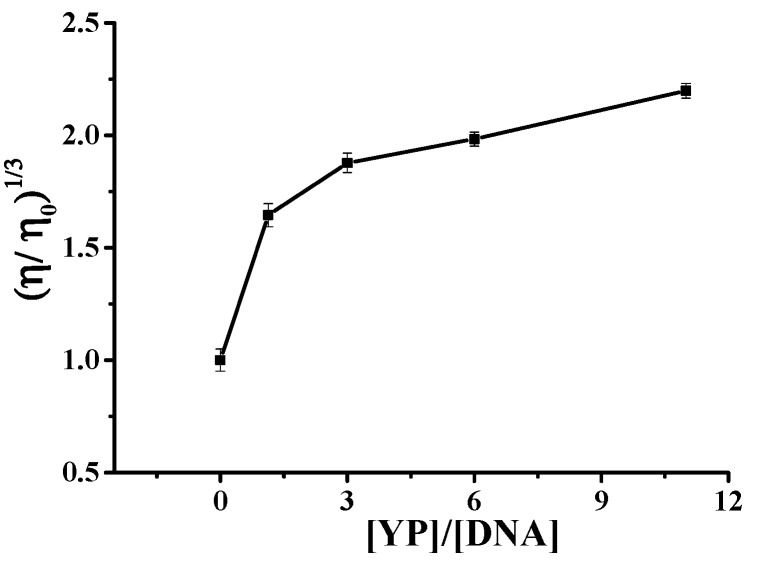
Relative viscosity of DNA–YP complexes in the presence of different YP concentrations.

Figure 6 shows the plot of (η/η_0_)^1/3^ versus *r* for the binding between YP and ctDNA. Results clearly showed that YP was intercalated between adjacent DNA base pairs, resulting in an extension of the DNA helix and a corresponding increase in DNA viscosity. The increase in viscosity became more obvious with increasing *r* (*r =* [YP]/[DNA]), providing further evidence for the intercalation reaction. It was speculated that YP and DNA first had an electrostatic reaction, and then they combined via an intercalation reaction.

### 2.5. Protection Against ·OH-Induced DNA Damage

**Figure 7:**
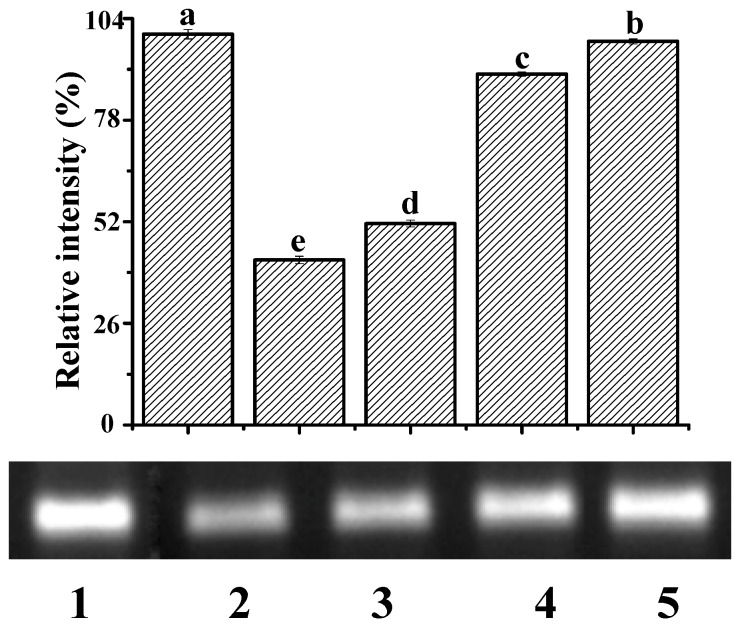
Protective effect of YP against ctDNA damage: Lane 1, DNA; Lane 2, DNA + ·OH; Lane 3, DNA + ·OH + 0.05 mg/mL YP; Lane 4, DNA + ·OH + 0.11 mg/mL YP; and Lane 5, DNA + ·OH + 0.17 mg/mL YP. Bar chart presents relative intensity of DNA band with respect to Lane 1 (DNA only).

Among reactive oxygen species, ∙OH is the most active in terms of DNA damage [33]. The ∙OH induced by UV irradiation damages the architecture of the DNA helix, causing the electrophoretic band of DNA to diminish. Figure 7 shows the electrophoretogram of DNA in the presence and absence of ∙OH and YP. The addition of ∙OH decreased the DNA band by 57.78% (Lane 2) compared to DNA alone (Lane 1). In the presence of both ∙OH and YP, the retention percentages of the DNA band were 51.54%, 89.78%, and 98.16% when YP concentrations were 0.05, 0.11, and 0.17 mg/mL, respectively. Thus, YP effectively protected DNA from damage induced by ∙OH in a dose-dependent manner. This protective capacity likely originates from the radical scavenging activity of YP and the stable structure of the DNA–YP complexes. YP and ctDNA were close to each other due to the unstable electrostatic binding during the initial reaction steps. Subsequent intercalative binding reduced the exposure of reactive groups in the DNA–YP complex, preventing ∙OH from damaging the helical architecture of the DNA–YP complex. Results agree with a previous study showing that the interaction of isoeugenol with ctDNA protects the DNA from oxidative damage [31].

### 2.6. Hydroxyl Radical Scavenging Activity

**Figure 8:**
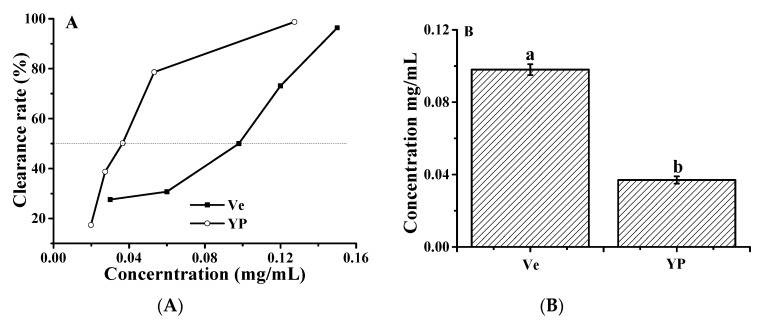
Hydroxyl radical scavenging activity of YP: (**A**) clearance rate as a function of YP or vitamin E (Ve) concentration and (**B**) IC_50_ values of YP and Ve.

We used vitamin E as a reference to compare the scavenging capacity of ∙OH in the YP. The above results indicate that YP was able to inhibit ∙OH-induced DNA damage in reaction mixtures containing ctDNA and H_2_O_2_. The ∙OH scavenging activity of YP and vitamin E increased with increasing concentrations of YP and vitamin E. YP exhibited ∙OH scavenging activity at low concentrations, and the IC_50_ value of YP (0.098 ± 0.032 mg/mL) was 12.35 times lower than that of vitamin E (1.21 ± 0.054 μg/mL; Figure 8). The protective effect of YP on DNA was due to the higher free radical scavenging activity, which reduced the amount of ∙OH and protected the DNA–YP complex from oxidative damage. Cyanidin-3-glucoside has a similar effect on protecting DNA from oxidative damage by ∙OH [34].

## 3. Discussion

Conventionally utilized natural pigments have attracted people’s attention for application in a variety of drugs, given they are medically proven to be therapeutically effective in increasing resistance to various diseases. The active oxygen species induced by oxidative stress in living cells cause damage to cellular macromolecules such as proteins, lipids, and DNA [35]. DNA, as the sensitive cellular target of those active radicals, always produces cumulative mutations due to the disrupted intracellular balance of active radicals. Furthermore, radicals were confirmed as an important probe of various degenerative diseases, such as cancer and aging [36]. Studies of the binding between YP and DNA are useful to understand the reaction mechanism and guide the direction of the application and design of new and more available drugs that act on DNA. The characteristics of YP binding to ctDNA were investigated by UV–Vis and fluorescence spectroscopic techniques. The YP exhibited a strong intercalative binding ability to form the YP–DNA complex. 

On the above basis, in order to investigate the mechanism of the DNA protection activity of YP, antioxidant activities were measured by assessing the capacity of YP to scavenge ∙OH. The absorbance of ∙OH in solution decreased upon receiving free radical atoms after reacting with an antioxidant compound. Results indicated that the YP was bound to DNA in an intercalative way. Above all, the protective effect of YP on DNA was due to its high free radical scavenging activity. Furthermore, changes in the space steric hindrance of ctDNA by the intercalation between YP and ctDNA might inhibit the damage to DNA by hydroxyl radicals. Although it was reported that small-molecule compounds {Na_5_[PMo_10_V_2_O_40_]·nH_2_O} could damage ctDNA through groove binding with ctDNA [37], there were also reports that the naturally formed pigment, cyanidin-3-glucoside, could protect ctDNA from hydroxyl radicals after intercalating with ctDNA [34], which was in agreement with our results. Whether the intercalative binding capacity of YP was related to the inhibition of ∙OH in DNA–YP complexes needs to be further researched.

## 4. Materials and Methods 

### 4.1. Materials and Instruments

All chemicals used within this experiment were of analytical quality and used without any further purification.

Type I fibrous ctDNA was purchased from Solarbio Science and Technology Co., Ltd. (Beijing, China). NR was obtained from Sinopharm Chemical Reagent Co., Ltd. (Shanghai, China). NaCl was purchased from Beijing Chemical Plant (Beijing, China). Redistilled water for all experiments was provided by a tridistilled evaporator (Model SZ-97A, Yarong Co., Ltd., Shanghai, China). Anhydrous ethanol, methanol, and acetonitrile were obtained from Dima Technologies (Beijing, China). Tris Borate–EDTA buffer solution (TBE, 10X) was purchased from Sigma (Santa Clara, CA, USA).

### 4.2. Methods

#### 4.2.1. Preparation of YP Extract

Yellow freshly cut yam (Figure 1A) and methanol were mixed in a 1:1 (m:v) ratio and soaked for 2 h. After filtration using filter paper, the methanol was removed by rotary evaporation. The resultant extract was separated by liquid chromatography using an Amberlite XAD-7 column (60 × 1.6 cm; Sigma, Santa Clara, CA, USA) on an AKTA explorer chromatography system (GE, Fairfield, CT, USA). The injection volume was 250 mL, and the column was washed with water (5 mL/min) to remove sugar and protein until electrical conductivity became stable. Subsequently, the pigment was eluted with methanol, and the eluent with an absorption peak at 410 nm was collected, concentrated by rotary evaporation (BUCHI R-215, Flawil, Switzerland), and then filtered using a 0.22 mm syringe filter. 

The purified sample was further cleaned using Sep-Pak C_18_ solid-phase extraction (SPE) cartridge that had been consecutively conditioned with 10 times the column volume of methanol and 10 times the column volume of water. A 1.5 mL aliquot was added to the SPE cartridge and eluted with 45 mL of water to remove impurities. The yellow pigment was then eluted with methanol. The methanol eluent was collected and concentrated by rotary evaporation. The final yellow powder was defined as YP.

#### 4.2.2. UV−Vis Spectroscopy

YP (0.9 mg/mL) of 0, 50, 60, and 70 μL was added into DNA solution (4 mg/mL; 25 μL) and held for 30 min at a room temperature. The mixture was then scanned from 213 to 287 nm using a UV-1800 spectrophotometer (Shimadzu Corp., Kyoto, Japan) with a resolution of 0.5 nm.

#### 4.2.3. Fluorescence Spectroscopy

The fluorescence spectra of the samples were collected using a SpectraMax^®^ I3 spectrometer (Molecular Devices LLC, Wals, Austria). Briefly, YP (50 μL; 0, 0.9, 1.1, or 1.3 mg/mL) was mixed with DNA (0.9 mg/mL; 50 μL) and allowed to equilibrate for 30 min at room temperature. The fluorescence spectra were then recorded in the wavelength range of 415 to 725 nm with an excitation wavelength of 380 nm and slit width of 2 nm.

Solutions of ctDNA (50 μL; 0.1, 0.2, 0.3, and 0.4 mg/mL) were added to solutions of YP (50 μL; 0.9 mg/mL). After incubation at 25 °C for 30 min, fluorescence intensity was recorded in the range of 415–725 nm with a slit width of 2 nm.

Competitive-displacement analysis was carried out using the method of Wang et al. [25], with minor modifications. The well-known DNA binding probe NR [20] was employed to determine the binding mode. Specifically, NR (10 μL; 5 × 10^−3^ g/mL) was reacted with DNA (50 μL; 0.9 mg/mL) for 30 min at room temperature. YP (0, 0.55, 0.65, or 0.75 mg/mL) was then loaded into the DNA/NR mixture over 30 min. The fluorescence spectra of the resultant solutions were then collected from 515–750 nm at an excitation wavelength of 490 nm and a slit width of 2 nm using a Tris-HCl (pH: 7.0) buffer solution.

#### 4.2.4. Cationic Quenching by NaCl

The cationic quenching of DNA–YP complexes was carried out according to the method of Amado et al. [38], with slight modification. Briefly, YP (50 µL; 1.5 mg/mL) was mixed with DNA (50 µL; 0.9 mg/mL) and maintained at 25 °C for 30 min. NaCl solution (50 µL; 0, 0.2, 0.4, or 0.6 M) was added into the DNA–YP solution and allowed to react for 30 min. The solution was then analyzed by fluorescence spectroscopy with an excitation wavelength of 380 nm and an emission slit width of 2 nm.

#### 4.2.5. Viscosity Measurements

Apparent viscosity was measured using a controlled-stress rheometer (AR1500ex; TA, New Castle, DE, USA) on the basis of the method of Zhou et al. [39] with slight modification. Viscosity was measured at 25 °C using a parallel steel plate (diameter = 40 mm) with a 1 mm gap as shear rate increased from 0.1 to 100 s^−1^. One mL of ctDNA (0.9 mg/mL) was added to 1 mL of different concentrations of YP (0, 1.02, 2.7, 5.4, and 9.9 mg/mL), and then each solution was mixed with 1 mL of water. Flow time was measured using a digital stopwatch, and the average time calculated for 5 replicates was obtained to evaluate the viscosity of ctDNA alone and mixtures of ctDNA with different molar ratios of YP (*r* = [YP]/[ctDNA]). Viscosity (*η*) was calculated from the observed flow time of the ctDNA-containing solution (*t*) and corrected using the flow time of the buffer solution (*t*_0_) using equation *η* = (*t* − *t*_0_)/*t*_0_. The obtained data were plotted as (*η*/*η*_0_)^1/3^ versus *r*, where *η* represents the viscosity of ctDNA in the presence of YP, and *η*_0_ is the viscosity in the buffer solution without YP.

#### 4.2.6. Protective Effect against DNA Damage

To study the protective effect of YP, DNA (0.15 mg/mL) was subjected to UV-induced hydroxyl radicals (∙OH) in the presence or absence of YP (0.05, 0.11, and 0.17 mg/mL) using the method of Li et al. [40], with modification. Hydrogen peroxide (H_2_O_2_; 50 μL; 1.5 × 10^−2^ mol·L^−1^) and DNA solution (100 μL) were mixed and irradiated with UV (130 Lux) in the presence or absence of YP for 2 min at room temperature. Resultant solutions (0.5 × TBE) were added to agarose gel (1%) containing goldview I of 0.4 μL at 55 °C. The volume ratio of sample solution to buffer solution was 4:2. The mixture (5 μL) was loaded onto a sample hole of 1% gel. Electrophoresis was conducted for approximately 30 min at 120 V (DYY-6C, Beijing Liuyi Co. Ltd., Beijing, China). DNA bands in the gel were imaged under a UV transilluminator [41].

#### 4.2.7. Hydroxyl Radical Scavenging Activity

∙OH scavenging activity was determined according to the method of Mathew et al. [42], with minor modifications. A working solution containing 0.5 mL FeCl_3_ (1 mM), 0.5 mL thylenediaminetetraacetic acid (1 mM), 0.5 mL deoxyribose (2.8 mM), and 1 mL sodium phosphate buffer (20 mM, pH 7.4) was prepared. Briefly, 200 μL of working solution was mixed with the same volume of extracts containing different concentrations. Before the reaction began, 20 μL of 1 mM ascorbic acid and 100 μL of 20 mM H_2_O_2_ were added and kept at 37 °C for 1 h. After incubation, 2.8% trichloroacetic acid (200 μL) was added to terminate the reaction in a water bath at 55 °C for 20 min. Subsequently, 1% 2-thiobarbituric acid (200 μL) was added, followed by heating at 100 °C for 30 min. The mixture supernatant was obtained after cooling and centrifugation. The absorbance of the supernatant containing YP (*A*_sample_) and buffer only (*A*_blank_) was measured at 532 nm. The protective effect was then calculated as a percentage as follows: protective effect (%) = (*A*_blank_ − *A*_sample_)/*A*_blank_ × 100, where *A* was the absorption value of the sample. The half-maximal inhibitory concentration (IC_50_) of ∙OH was determined by linear regression of the plot of the percentage of remaining ∙OH against the sample concentration.

## 5. Conclusions

Natural pigments have attracted attention for application in numerous drugs because of their therapeutic effectiveness against various diseases. Upon mixing, YP and DNA are initially attracted electrostatically. Subsequently, YP intercalates into DNA to form DNA–YP complexes, as confirmed by UV–Vis and fluorescence spectroscopy. YP exhibited a protective effect against DNA damage due to its free radical scavenging ability. Whether the intercalative binding capacity of YP is related to the inhibition of ∙OH in DNA–YP complexes requires further study.

## 6. Highlight

— The interaction of yam yellow pigment with calf thymus DNA was investigated.
— DNA bound yellow pigment through intercalative binding.
— Yam yellow pigment protected DNA from damage by hydroxyl radicals.
